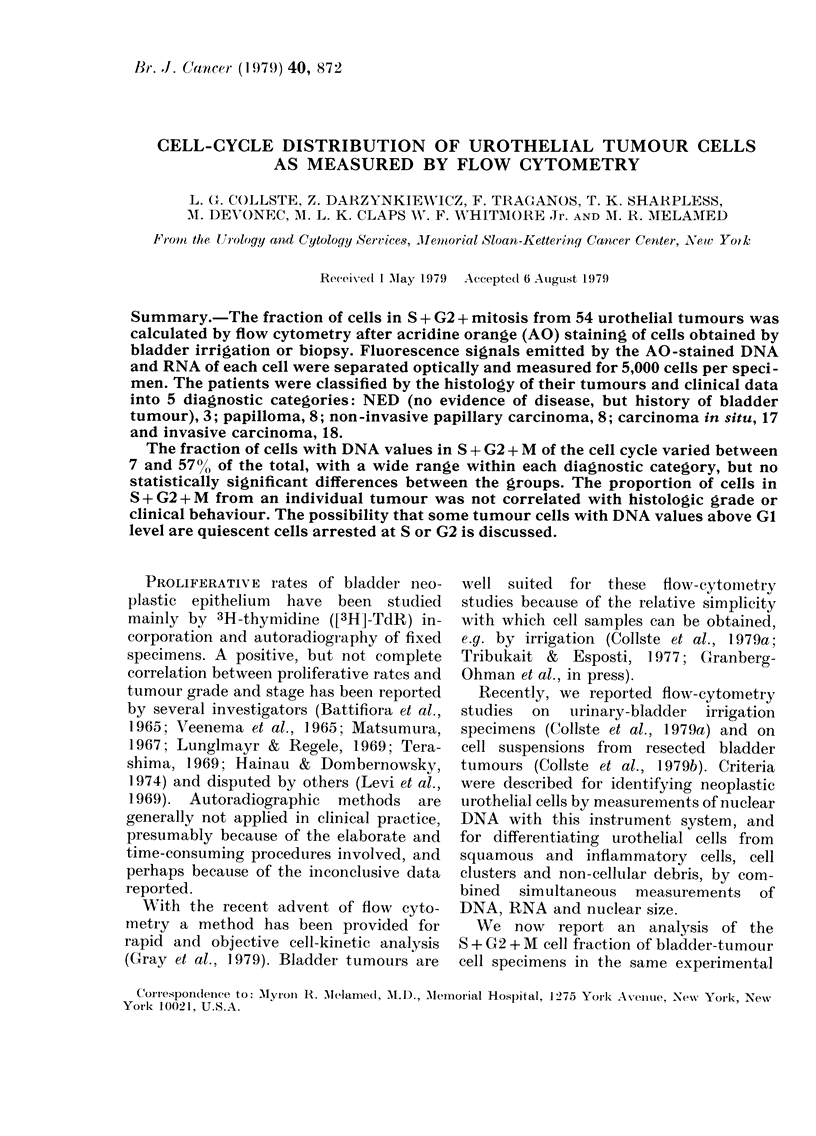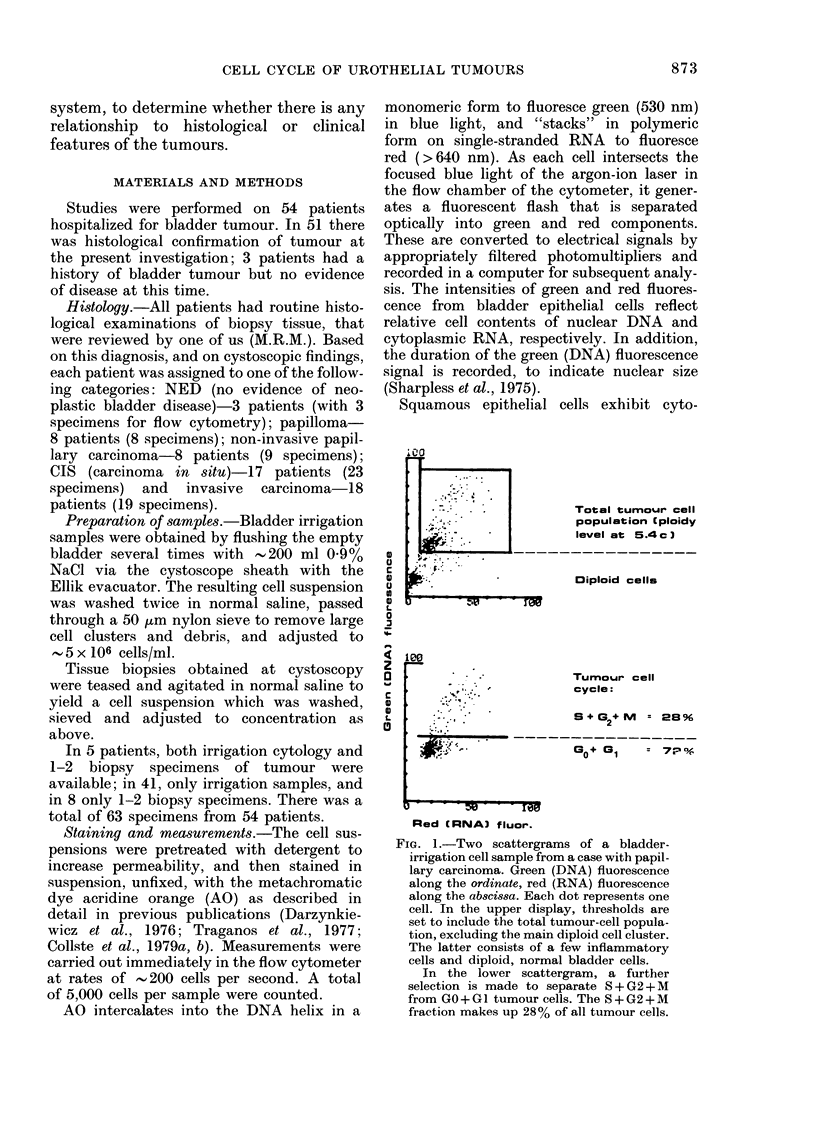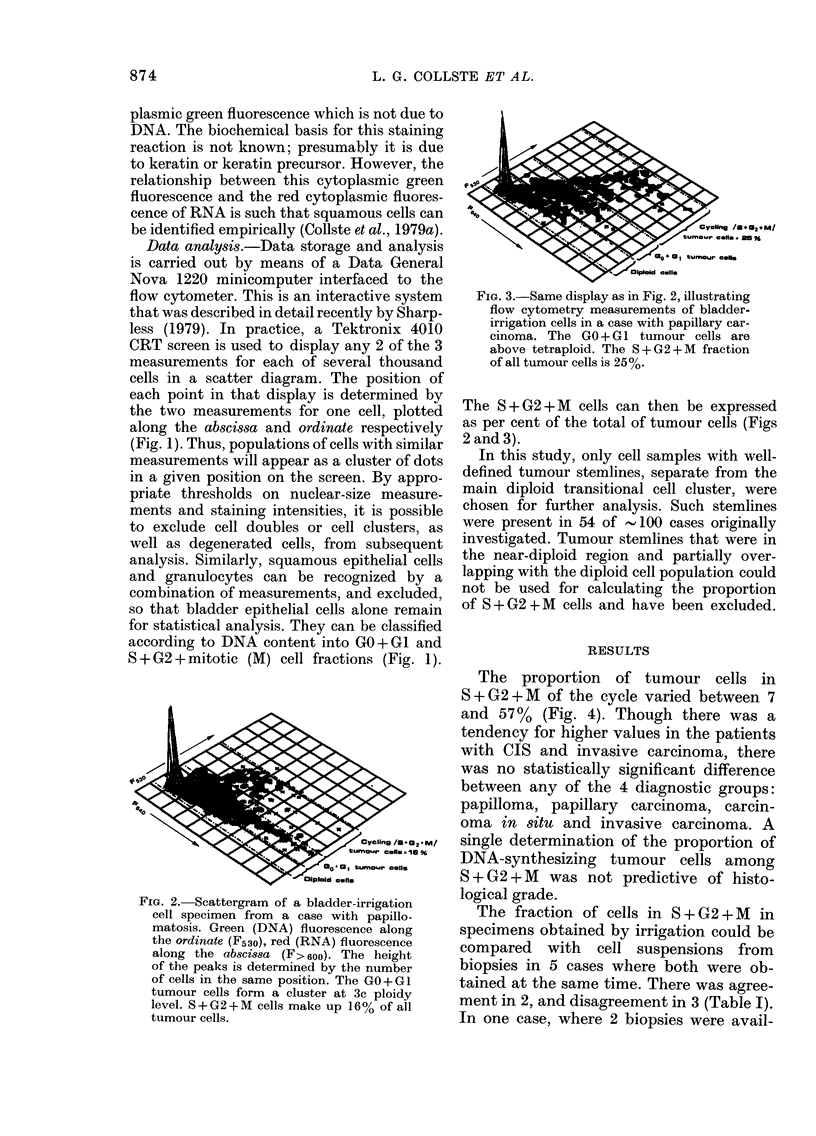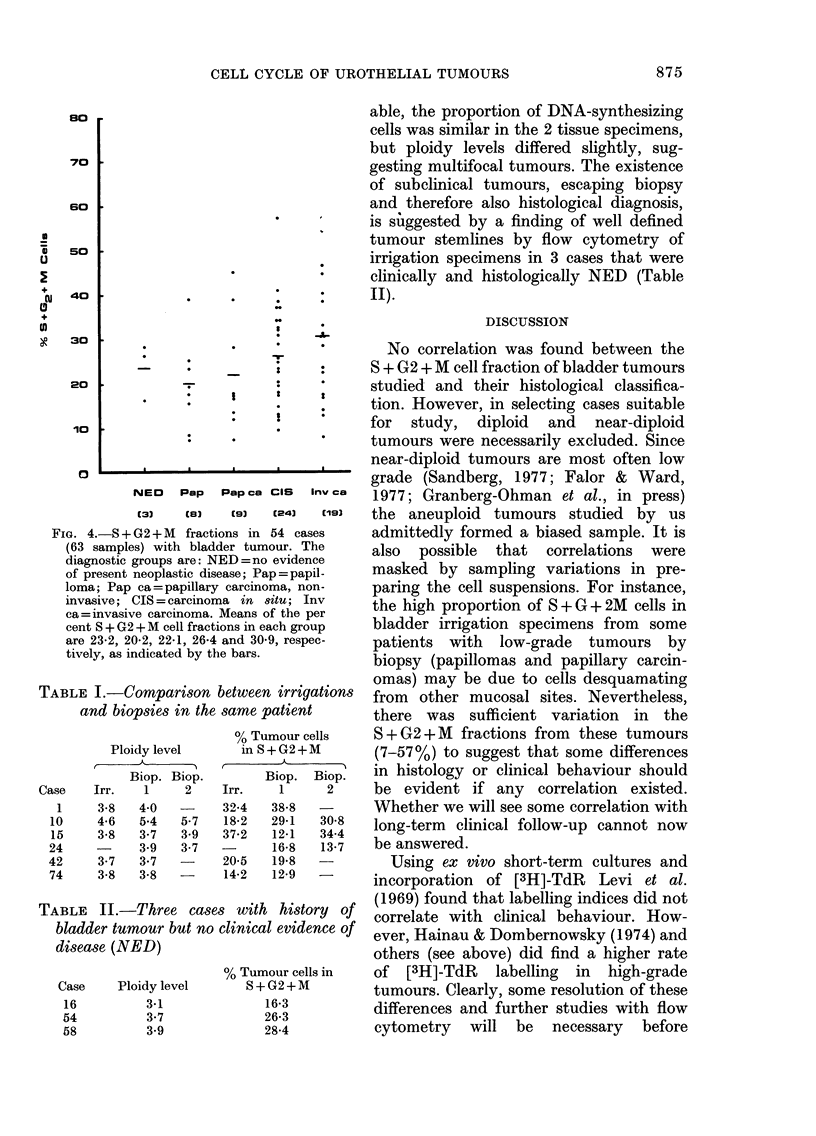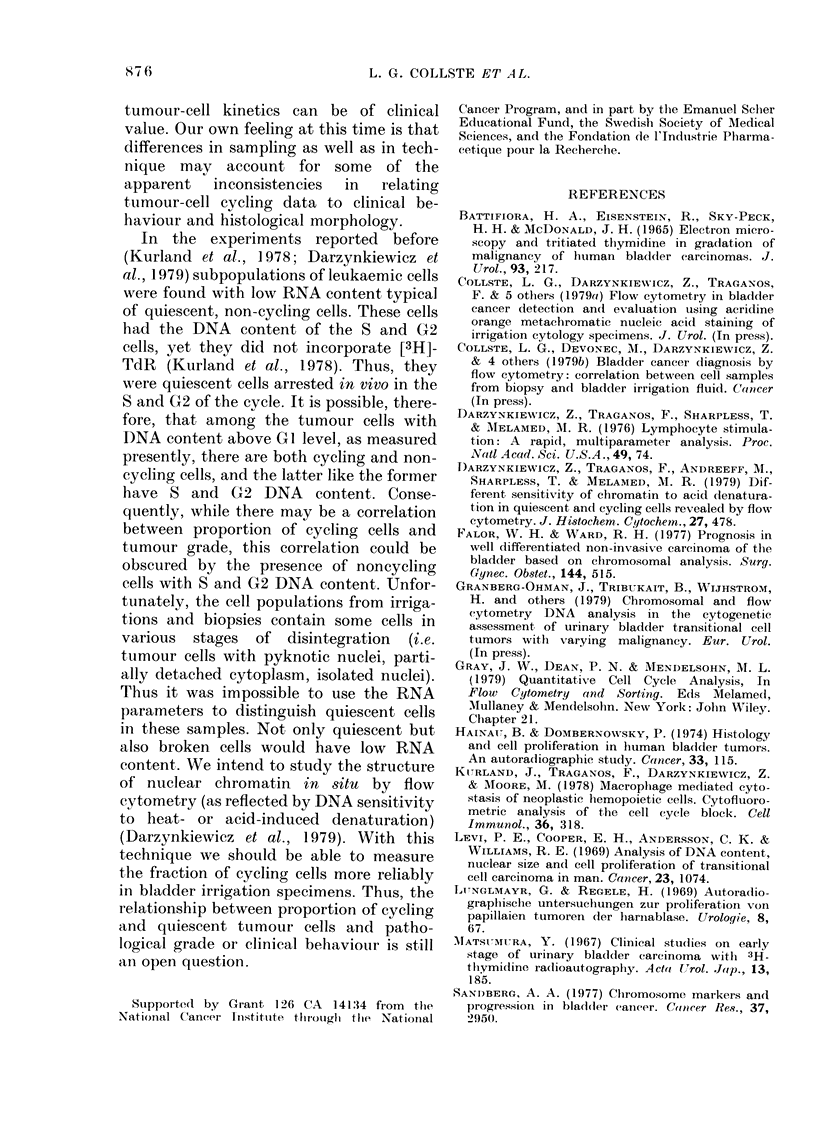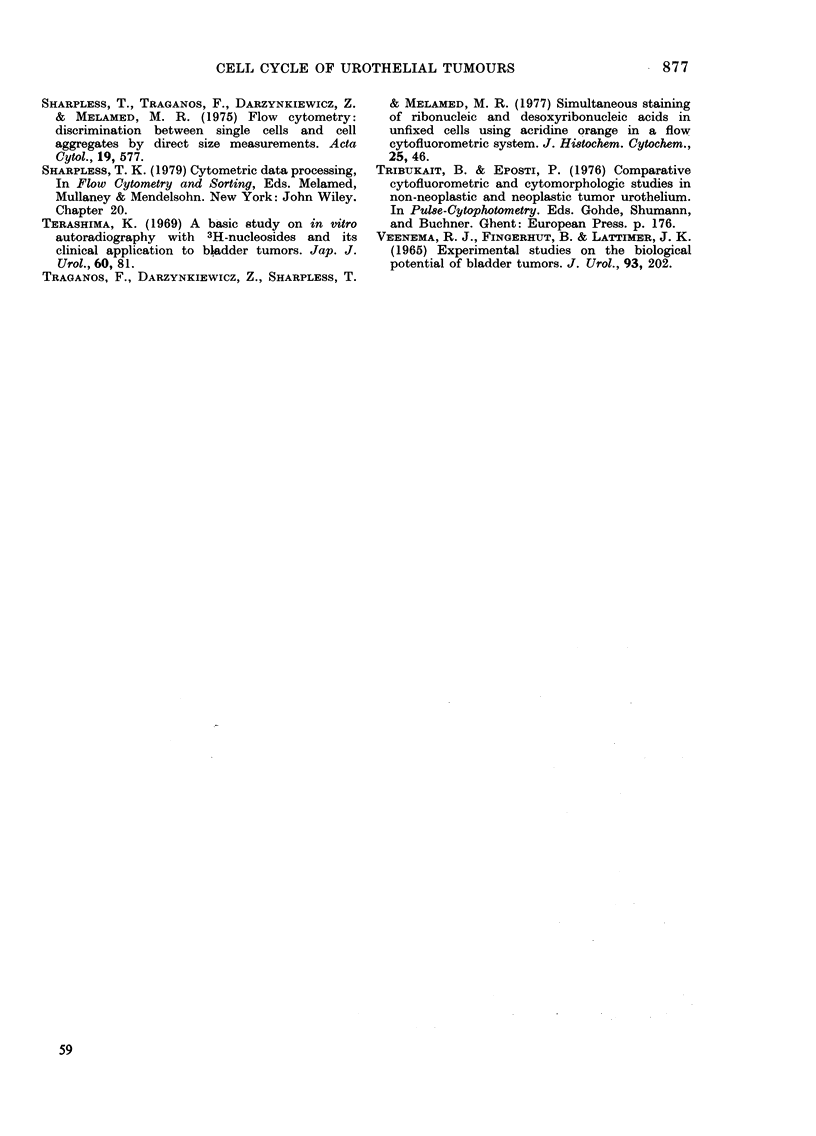# Cell-cycle distribution of urothelial tumour cells as measured by flow cytometry.

**DOI:** 10.1038/bjc.1979.279

**Published:** 1979-12

**Authors:** L. G. Collste, Z. Darzynkiewicz, F. Traganos, T. K. Sharpless, M. Devonec, M. L. Claps, W. F. Whitmore, M. R. Melamed

## Abstract

The fraction of cells in S + G2 + mitosis from 54 urothelial tumours was calculated by flow cytometry after acridine orange (AO) staining of cells obtained by bladder irrigation or biopsy. Fluorescence signals emitted by the AO-stained DNA and RNA of each cell were separated optically and measured for 5,000 cells per specimen. The patients were classified by the histology of their tumours and clinical data into 5 diagnostic categories: NED (no evidence of disease, but history of bladder tumour), 3; papilloma, 8; non-invasive papillary carcinoma, 8; carcinoma in situ, 17 and invasive carcinoma, 18. The fraction of cells with DNA values in S + G2 + M of the cell cycle varied between 7 and 57% of the total, with a wide range within each diagnostic category, but no statistically significant differences between the groups. The proportion of cells in S + G2 + M from an individual tumour was not correlated with histologic grade or clinical behaviour. The possibility that some tumour cells with DNA values above G1 level are quiescent cells arrested at S or G2 is discussed.


*Br. J. Cancer* (1979) **40**, 872

### CELL-CYCLE DISTRIBUTION OF UROTHELIAL TUMOUR CELLS AS MEASURED BY FLOW CYTOMETRY

L. G. COLLSTE, Z. DARZYNKIEWICZ, F. TRAGANOS, T. K. SHARPLESS, M. DEVONEC, M. L. K. CLAPS W. F. WHITMORE Jr. AND M. R. MELAMED

*From the Urology and Cytology Services, Memorial Sloan-Kettering Cancer Center, New York*

Received 1 May 1979 Accepted 6 August 1979

**Summary.—The fraction of cells in S + G2 + mitosis from 54 urothelial tumours was calculated by flow cytometry after acridine orange (AO) staining of cells obtained by bladder irrigation or biopsy. Fluorescence signals emitted by the AO-stained DNA and RNA of each cell were separated optically and measured for 5,000 cells per specimen. The patients were classified by the histology of their tumours and clinical data into 5 diagnostic categories: NED (no evidence of disease, but history of bladder tumour), 3; papilloma, 8; non-invasive papillary carcinoma, 8; carcinoma *in situ*, 17 and invasive carcinoma, 18.**

**The fraction of cells with DNA values in S + G2 + M of the cell cycle varied between 7 and 57% of the total, with a wide range within each diagnostic category, but no statistically significant differences between the groups. The proportion of cells in S + G2 + M from an individual tumour was not correlated with histologic grade or clinical behaviour. The possibility that some tumour cells with DNA values above G1 level are quiescent cells arrested at S or G2 is discussed.**

Proliferative rates of bladder neoplastic epithelium have been studied mainly by $^3$H-thymidine ([$^3$H]-TdR) incorporation and autoradiography of fixed specimens. A positive, but not complete correlation between proliferative rates and tumour grade and stage has been reported by several investigators (Battifiora *et al.*, 1965; Veenema *et al.*, 1965; Matsumura, 1967; Lunglmayr & Regele, 1969; Terashima, 1969; Hainau & Dombernowsky, 1974) and disputed by others (Levi *et al.*, 1969). Autoradiographic methods are generally not applied in clinical practice, presumably because of the elaborate and time-consuming procedures involved, and perhaps because of the inconclusive data reported.

With the recent advent of flow cytometry a method has been provided for rapid and objective cell-kinetic analysis (Gray *et al.*, 1979). Bladder tumours are well suited for these flow-cytometry studies because of the relative simplicity with which cell samples can be obtained, *e.g.* by irrigation (Collste *et al.*, 1979*a*; Tribukait & Esposti, 1977; Granberg-Ohman *et al.*, in press).

Recently, we reported flow-cytometry studies on urinary-bladder irrigation specimens (Collste *et al.*, 1979*a*) and on cell suspensions from resected bladder tumours (Collste *et al.*, 1979*b*). Criteria were described for identifying neoplastic urothelial cells by measurements of nuclear DNA with this instrument system, and for differentiating urothelial cells from squamous and inflammatory cells, cell clusters and non-cellular debris, by combined simultaneous measurements of DNA, RNA and nuclear size.

We now report an analysis of the S + G2 + M cell fraction of bladder-tumour cell specimens in the same experimental

Correspondence to: Myron R. Melamed, M.D., Memorial Hospital, 1275 York Avenue, New York, New York 10021, U.S.A.

system, to determine whether there is any relationship to histological or clinical features of the tumours.

### MATERIALS AND METHODS

Studies were performed on 54 patients hospitalized for bladder tumour. In 51 there was histological confirmation of tumour at the present investigation; 3 patients had a history of bladder tumour but no evidence of disease at this time.

*Histology*.—All patients had routine histological examinations of biopsy tissue, that were reviewed by one of us (M.R.M.). Based on this diagnosis, and on cystoscopic findings, each patient was assigned to one of the following categories: NED (no evidence of neoplastic bladder disease)—3 patients (with 3 specimens for flow cytometry); papilloma—8 patients (8 specimens); non-invasive papillary carcinoma—8 patients (9 specimens); CIS (carcinoma *in situ*)—17 patients (23 specimens) and invasive carcinoma—18 patients (19 specimens).

*Preparation of samples*.—Bladder irrigation samples were obtained by flushing the empty bladder several times with ~200 ml 0·9% NaCl via the cystoscope sheath with the Ellik evacuator. The resulting cell suspension was washed twice in normal saline, passed through a 50 μm nylon sieve to remove large cell clusters and debris, and adjusted to ~5 x $10^6$ cells/ml.

Tissue biopsies obtained at cystoscopy were teased and agitated in normal saline to yield a cell suspension which was washed, sieved and adjusted to concentration as above.

In 5 patients, both irrigation cytology and 1–2 biopsy specimens of tumour were available; in 41, only irrigation samples, and in 8 only 1–2 biopsy specimens. There was a total of 63 specimens from 54 patients.

*Staining and measurements*.—The cell suspensions were pretreated with detergent to increase permeability, and then stained in suspension, unfixed, with the metachromatic dye acridine orange (AO) as described in detail in previous publications (Darzynkiewicz *et al.*, 1976; Traganos *et al.*, 1977; Collste *et al.*, 1979*a*, *b*). Measurements were carried out immediately in the flow cytometer at rates of ~200 cells per second. A total of 5,000 cells per sample were counted.

AO intercalates into the DNA helix in a monomeric form to fluoresce green (530 nm) in blue light, and "stacks" in polymeric form on single-stranded RNA to fluoresce red (>640 nm). As each cell intersects the focused blue light of the argon-ion laser in the flow chamber of the cytometer, it generates a fluorescent flash that is separated optically into green and red components. These are converted to electrical signals by appropriately filtered photomultipliers and recorded in a computer for subsequent analysis. The intensities of green and red fluorescence from bladder epithelial cells reflect relative cell contents of nuclear DNA and cytoplasmic RNA, respectively. In addition, the duration of the green (DNA) fluorescence signal is recorded, to indicate nuclear size (Sharpless *et al.*, 1975).

Squamous epithelial cells exhibit cyto-

FIG. 1.—Two scattergrams of a bladder-irrigation cell sample from a case with papillary carcinoma. Green (DNA) fluorescence along the *ordinate*, red (RNA) fluorescence along the *abscissa*. Each dot represents one cell. In the upper display, thresholds are set to include the total tumour-cell population, excluding the main diploid cell cluster. The latter consists of a few inflammatory cells and diploid, normal bladder cells.

In the lower scattergram, a further selection is made to separate S+G2+M from G0+G1 tumour cells. The S+G2+M fraction makes up 28% of all tumour cells.

plasmic green fluorescence which is not due to DNA. The biochemical basis for this staining reaction is not known; presumably it is due to keratin or keratin precursor. However, the relationship between this cytoplasmic green fluorescence and the red cytoplasmic fluorescence of RNA is such that squamous cells can be identified empirically (Collste *et al.*, 1979*a*).

*Data analysis*.—Data storage and analysis is carried out by means of a Data General Nova 1220 minicomputer interfaced to the flow cytometer. This is an interactive system that was described in detail recently by Sharpless (1979). In practice, a Tektronix 4010 CRT screen is used to display any 2 of the 3 measurements for each of several thousand cells in a scatter diagram. The position of each point in that display is determined by the two measurements for one cell, plotted along the *abscissa* and *ordinate* respectively (Fig. 1). Thus, populations of cells with similar measurements will appear as a cluster of dots in a given position on the screen. By appropriate thresholds on nuclear-size measurements and staining intensities, it is possible to exclude cell doubles or cell clusters, as well as degenerated cells, from subsequent analysis. Similarly, squamous epithelial cells and granulocytes can be recognized by a combination of measurements, and excluded, so that bladder epithelial cells alone remain for statistical analysis. They can be classified according to DNA content into G0+G1 and S+G2+mitotic (M) cell fractions (Fig. 1).

FIG. 2.—Scattergram of a bladder-irrigation cell specimen from a case with papillomatosis. Green (DNA) fluorescence along the *ordinate* ($F_{530}$), red (RNA) fluorescence along the *abscissa* ($F_{>600}$). The height of the peaks is determined by the number of cells in the same position. The G0+G1 tumour cells form a cluster at 3c ploidy level. S+G2+M cells make up 16% of all tumour cells.

FIG. 3.—Same display as in Fig. 2, illustrating flow cytometry measurements of bladder-irrigation cells in a case with papillary carcinoma. The G0+G1 tumour cells are above tetraploid. The S+G2+M fraction of all tumour cells is 25%.

The S+G2+M cells can then be expressed as per cent of the total of tumour cells (Figs 2 and 3).

In this study, only cell samples with well-defined tumour stemlines, separate from the main diploid transitional cell cluster, were chosen for further analysis. Such stemlines were present in 54 of ~100 cases originally investigated. Tumour stemlines that were in the near-diploid region and partially overlapping with the diploid cell population could not be used for calculating the proportion of S+G2+M cells and have been excluded.

### RESULTS

The proportion of tumour cells in S+G2+M of the cycle varied between 7 and 57% (Fig. 4). Though there was a tendency for higher values in the patients with CIS and invasive carcinoma, there was no statistically significant difference between any of the 4 diagnostic groups: papilloma, papillary carcinoma, carcinoma *in situ* and invasive carcinoma. A single determination of the proportion of DNA-synthesizing tumour cells among S+G2+M was not predictive of histological grade.

The fraction of cells in S+G2+M in specimens obtained by irrigation could be compared with cell suspensions from biopsies in 5 cases where both were obtained at the same time. There was agreement in 2, and disagreement in 3 (Table I). In one case, where 2 biopsies were avail-

FIG. 4.—S+G2+M fractions in 54 cases (63 samples) with bladder tumour. The diagnostic groups are: NED=no evidence of present neoplastic disease; Pap=papilloma; Pap ca=papillary carcinoma, non-invasive; CIS=carcinoma *in situ*; Inv ca=invasive carcinoma. Means of the per cent S+G2+M cell fractions in each group are 23·2, 20·2, 22·1, 26·4 and 30·9, respectively, as indicated by the bars.

TABLE I.—*Comparison between irrigations and biopsies in the same patient*

| | Ploidy level | | | % Tumour cells in S+G2+M | | |
|---|---|---|---|---|---|---|
| Case | Irr. | Biop. 1 | Biop. 2 | Irr. | Biop. 1 | Biop. 2 |
| 1 | 3·8 | 4·0 | — | 32·4 | 38·8 | — |
| 10 | 4·6 | 5·4 | 5·7 | 18·2 | 29·1 | 30·8 |
| 15 | 3·8 | 3·7 | 3·9 | 37·2 | 12·1 | 34·4 |
| 24 | — | 3·9 | 3·7 | — | 16·8 | 13·7 |
| 42 | 3·7 | 3·7 | — | 20·5 | 19·8 | — |
| 74 | 3·8 | 3·8 | — | 14·2 | 12·9 | — |

TABLE II.—*Three cases with history of bladder tumour but no clinical evidence of disease (NED)*

| Case | Ploidy level | % Tumour cells in S+G2+M |
|---|---|---|
| 16 | 3·1 | 16·3 |
| 54 | 3·7 | 26·3 |
| 58 | 3·9 | 28·4 |

able, the proportion of DNA-synthesizing cells was similar in the 2 tissue specimens, but ploidy levels differed slightly, suggesting multifocal tumours. The existence of subclinical tumours, escaping biopsy and therefore also histological diagnosis, is suggested by a finding of well defined tumour stemlines by flow cytometry of irrigation specimens in 3 cases that were clinically and histologically NED (Table II).

### DISCUSSION

No correlation was found between the S+G2+M cell fraction of bladder tumours studied and their histological classification. However, in selecting cases suitable for study, diploid and near-diploid tumours were necessarily excluded. Since near-diploid tumours are most often low grade (Sandberg, 1977; Falor & Ward, 1977; Granberg-Ohman *et al.*, in press) the aneuploid tumours studied by us admittedly formed a biased sample. It is also possible that correlations were masked by sampling variations in preparing the cell suspensions. For instance, the high proportion of S+G+2M cells in bladder irrigation specimens from some patients with low-grade tumours by biopsy (papillomas and papillary carcinomas) may be due to cells desquamating from other mucosal sites. Nevertheless, there was sufficient variation in the S+G2+M fractions from these tumours (7–57%) to suggest that some differences in histology or clinical behaviour should be evident if any correlation existed. Whether we will see some correlation with long-term clinical follow-up cannot now be answered.

Using *ex vivo* short-term cultures and incorporation of [$^3$H]-TdR Levi *et al.* (1969) found that labelling indices did not correlate with clinical behaviour. However, Hainau & Dombernowsky (1974) and others (see above) did find a higher rate of [$^3$H]-TdR labelling in high-grade tumours. Clearly, some resolution of these differences and further studies with flow cytometry will be necessary before tumour-cell kinetics can be of clinical value. Our own feeling at this time is that differences in sampling as well as in technique may account for some of the apparent inconsistencies in relating tumour-cell cycling data to clinical behaviour and histological morphology.

In the experiments reported before (Kurland *et al.*, 1978; Darzynkiewicz *et al.*, 1979) subpopulations of leukaemic cells were found with low RNA content typical of quiescent, non-cycling cells. These cells had the DNA content of the S and G2 cells, yet they did not incorporate [$^3$H]-TdR (Kurland *et al.*, 1978). Thus, they were quiescent cells arrested *in vivo* in the S and G2 of the cycle. It is possible, therefore, that among the tumour cells with DNA content above G1 level, as measured presently, there are both cycling and non-cycling cells, and the latter like the former have S and G2 DNA content. Consequently, while there may be a correlation between proportion of cycling cells and tumour grade, this correlation could be obscured by the presence of noncycling cells with S and G2 DNA content. Unfortunately, the cell populations from irrigations and biopsies contain some cells in various stages of disintegration (*i.e.* tumour cells with pyknotic nuclei, partially detached cytoplasm, isolated nuclei). Thus it was impossible to use the RNA parameters to distinguish quiescent cells in these samples. Not only quiescent but also broken cells would have low RNA content. We intend to study the structure of nuclear chromatin *in situ* by flow cytometry (as reflected by DNA sensitivity to heat- or acid-induced denaturation) (Darzynkiewicz *et al.*, 1979). With this technique we should be able to measure the fraction of cycling cells more reliably in bladder irrigation specimens. Thus, the relationship between proportion of cycling and quiescent tumour cells and pathological grade or clinical behaviour is still an open question.

Supported by Grant 126 CA 14134 from the National Cancer Institute through the National Cancer Program, and in part by the Emanuel Scher Educational Fund, the Swedish Society of Medical Sciences, and the Fondation de l'Industrie Pharmacetique pour la Recherche.

### REFERENCES

BATTIFIORA, H. A., EISENSTEIN, R., SKY-PECK, H. H. & MCDONALD, J. H. (1965) Electron microscopy and tritiated thymidine in gradation of malignancy of human bladder carcinomas. *J. Urol.*, **93**, 217.

COLLSTE, L. G., DARZYNKIEWICZ, Z., TRAGANOS, F. & 5 others (1979*a*) Flow cytometry in bladder cancer detection and evaluation using acridine orange metachromatic nucleic acid staining of irrigation cytology specimens. *J. Urol.* (In press).

COLLSTE, L. G., DEVONEC, M., DARZYNKIEWICZ, Z. & 4 others (1979*b*) Bladder cancer diagnosis by flow cytometry: correlation between cell samples from biopsy and bladder irrigation fluid. *Cancer* (In press).

DARZYNKIEWICZ, Z., TRAGANOS, F., SHARPLESS, T. & MELAMED, M. R. (1976) Lymphocyte stimulation: A rapid, multiparameter analysis. *Proc. Natl Acad. Sci. U.S.A.*, **49**, 74.

DARZYNKIEWICZ, Z., TRAGANOS, F., ANDREEFF, M., SHARPLESS, T. & MELAMED, M. R. (1979) Different sensitivity of chromatin to acid denaturation in quiescent and cycling cells revealed by flow cytometry. *J. Histochem. Cytochem.*, **27**, 478.

FALOR, W. H. & WARD, R. H. (1977) Prognosis in well differentiated non-invasive carcinoma of the bladder based on chromosomal analysis. *Surg. Gynec. Obstet.*, **144**, 515.

GRANBERG-OHMAN, J., TRIBUKAIT, B., WIJHSTROM, H. and others (1979) Chromosomal and flow cytometry DNA analysis in the cytogenetic assessment of urinary bladder transitional cell tumors with varying malignancy. *Eur. Urol.* (In press).

GRAY, J. W., DEAN, P. N. & MENDELSOHN, M. L. (1979) Quantitative Cell Cycle Analysis, In *Flow Cytometry and Sorting.* Eds Melamed, Mullaney & Mendelsohn. New York: John Wiley. Chapter 21.

HAINAU, B. & DOMBERNOWSKY, P. (1974) Histology and cell proliferation in human bladder tumors. An autoradiographic study. *Cancer*, **33**, 115.

KURLAND, J., TRAGANOS, F., DARZYNKIEWICZ, Z. & MOORE, M. (1978) Macrophage mediated cytostasis of neoplastic hemopoietic cells. Cytofluorometric analysis of the cell cycle block. *Cell Immunol.*, **36**, 318.

LEVI, P. E., COOPER, E. H., ANDERSSON, C. K. & WILLIAMS, R. E. (1969) Analysis of DNA content, nuclear size and cell proliferation of transitional cell carcinoma in man. *Cancer*, **23**, 1074.

LUNGLMAYR, G. & REGELE, H. (1969) Autoradiographische untersuchungen zur proliferation von papillaien tumoren der harnablase. *Urologie*, **8**, 67.

MATSUMURA, Y. (1967) Clinical studies on early stage of urinary bladder carcinoma with $^3$H-thymidine radioautography. *Acta Urol. Jap.*, **13**, 185.

SANDBERG, A. A. (1977) Chromosome markers and progression in bladder cancer. *Cancer Res.*, **37**, 2950.

SHARPLESS, T., TRAGANOS, F., DARZYNKIEWICZ, Z. & MELAMED, M. R. (1975) Flow cytometry: discrimination between single cells and cell aggregates by direct size measurements. *Acta Cytol.*, **19**, 577.

SHARPLESS, T. K. (1979) Cytometric data processing, In *Flow Cytometry and Sorting*, Eds. Melamed, Mullaney & Mendelsohn. New York: John Wiley. Chapter 20.

TERASHIMA, K. (1969) A basic study on *in vitro* autoradiography with $^3$H-nucleosides and its clinical application to bladder tumors. *Jap. J. Urol.*, **60**, 81.

TRAGANOS, F., DARZYNKIEWICZ, Z., SHARPLESS, T. & MELAMED, M. R. (1977) Simultaneous staining of ribonucleic and desoxyribonucleic acids in unfixed cells using acridine orange in a flow cytofluorometric system. *J. Histochem. Cytochem.*, **25**, 46.

TRIBUKAIT, B. & EPOSTI, P. (1976) Comparative cytofluorometric and cytomorphologic studies in non-neoplastic and neoplastic tumor urothelium. In *Pulse-Cytophotometry*. Eds. Gohde, Shumann, and Buchner. Ghent: European Press. p. 176.

VEENEMA, R. J., FINGERHUT, B. & LATTIMER, J. K. (1965) Experimental studies on the biological potential of bladder tumors. *J. Urol.*, **93**, 202.